# The blood glucose-potassium ratio at admission predicts in-hospital mortality in patients with acute type A aortic dissection

**DOI:** 10.1038/s41598-023-42827-2

**Published:** 2023-09-21

**Authors:** Yaqin Chen, Yanchun Peng, Xuecui Zhang, Xiaoqin Liao, Jianlong Lin, Liangwan Chen, Yanjuan Lin

**Affiliations:** 1 School of Nursing, Fujian Medical University, Fuzhou, China; 2 Department of Nursing, Union Hospital of Fujian Medical University, No.29 Xinquan Road, Fuzhou, 350001 Fujian Province China; 3 Department of Cardiac Surgery, Union Hospital of Fujian Medical University, No.29 Xinquan Road, Fuzhou, 350001 Fujian Province China; 4 Department of Cardiac Surgery Nursing, Union Hospital of Fujian Medical University, Fuzhou, China

**Keywords:** Biomarkers, Cardiology, Medical research

## Abstract

Acute type A aortic dissection (ATAAD) is a serious cardiovascular emergency with high risk and mortality after surgery. Recent studies have shown that serum glucose-potassium ratio (GPR) is associated with the prognosis of cerebrovascular diseases. The purpose of this study was to investigate the relationship between GPR and in-hospital mortality in patients with ATAAD. From June 2019 to August 2021, we retrospectively analyzed the clinical data of 272 patients who underwent ATAAD surgery. According to the median value of GPR (1.74), the patients were divided into two groups. Univariate and multivariate logistic regression analysis were used to determine the risk factors of in-hospital mortality after ATAAD. In-hospital death was significantly more common in the high GPR group (> 1.74) (24.4% vs 13.9%; *P* = 0.027). The incidence of renal dysfunction in the low GPR group was significantly higher than that in the high GPR group (26.3% vs 14.8%: *P* = 0.019). After controlling for potential confounding variables and adjusting for multivariate logistic regression analysis, the results showed a high GPR (> 1.74) (AOR 4.70, 95% confidence interval (CI) 2.13–10.40; *P* =  < 0.001), lactic acid (AOR 1.14, 95% CI 1.03–1.26; *P* = 0.009), smokers (AOR 2.45, 95% CI 1.18–15.07; *P* = 0.039), mechanical ventilation (AOR 9.47, 95% CI 4.00–22.38; *P* =  < 0.001) was independent risk factor for in-hospital mortality in ATAAD patients, albumin (AOR 0.90, 95% CI 0.83–0.98; *P* = 0.014) was a protective factor for in-hospital prognosis. High GPR is a good predictor of in-hospital mortality after ATAAD surgery.

## Introduction

Acute type A aortic dissection (ATAAD) is a life-threatening disease, which usually requires emergency surgical intervention^1^. What is worrying is that the overall survival of patients with ATAAD after surgery is not optimistic. It has a poorer prognosis than acute type B dissection^2,3^, with approximately 18–30% of patients dying on admission^4,5^. Clinical outcomes of ATAAD patients vary widely^6^, and accurate risk stratification can influence further treatment (for example, choice of surgical type)^7^. Currently, predictive biomarkers for identifying the risk of death in ATAAD patients are important for risk stratification. Many studies have confirmed that inflammation and blood markers are related to the prognosis of ATAAD patients^8–10^. It is necessary to identify other simple biomarkers, and timely identification of high-risk patients will help improve the overall prognosis of ATAAD patients.

Blood glucose and potassium are two important blood indicators commonly used clinically, and their peripheral analysis is a simple and low-cost test. In the energy theory, glucose is the main energy source and plays a key role in maintaining cell metabolism^11^. Potassium ions mostly exist in intracellular fluid, which plays an important role in heart beating and maintaining kidney function. Studies have proved that there is a complex interaction between glucose and potassium in the human body^12^. Therefore, considering the potential joint effect of blood glucose and potassium, the blood gluose ratio (GPR) was introduced^13^. GPR has been proven to be an early prognostic factor for mortality in certain diseases, including ischemic stroke (IS), traumatic brain injury, and blunt abdominal trauma^13–15^. In intensive care unit (ICU) trauma patients, the variability of blood glucose and K^+^ (SDs) is a risk factor for adverse clinical outcomes, and blood glucose is also a risk factor for the prognosis of ATAAD^16^. However, the relationship between GPR at admission and postoperative clinical outcomes in ATAAD patients remains unclear. In view of the above situation, the clinical value of combined use of blood glucose and potassium for cardiovascular diseases still needs to be further explored.

Therefore, we aimed to investigate the association between GPR at admission and in-hospital mortality in ATAAD patients in a retrospective study. To provide a reference for better finding clinical indicators that are highly correlated with in-hospital mortality of ATAAD, which are economical and easy to detect, and have important clinical significance for reducing in-hospital mortality after ATAAD.

## Methods

### Patients

**Figure 1:**
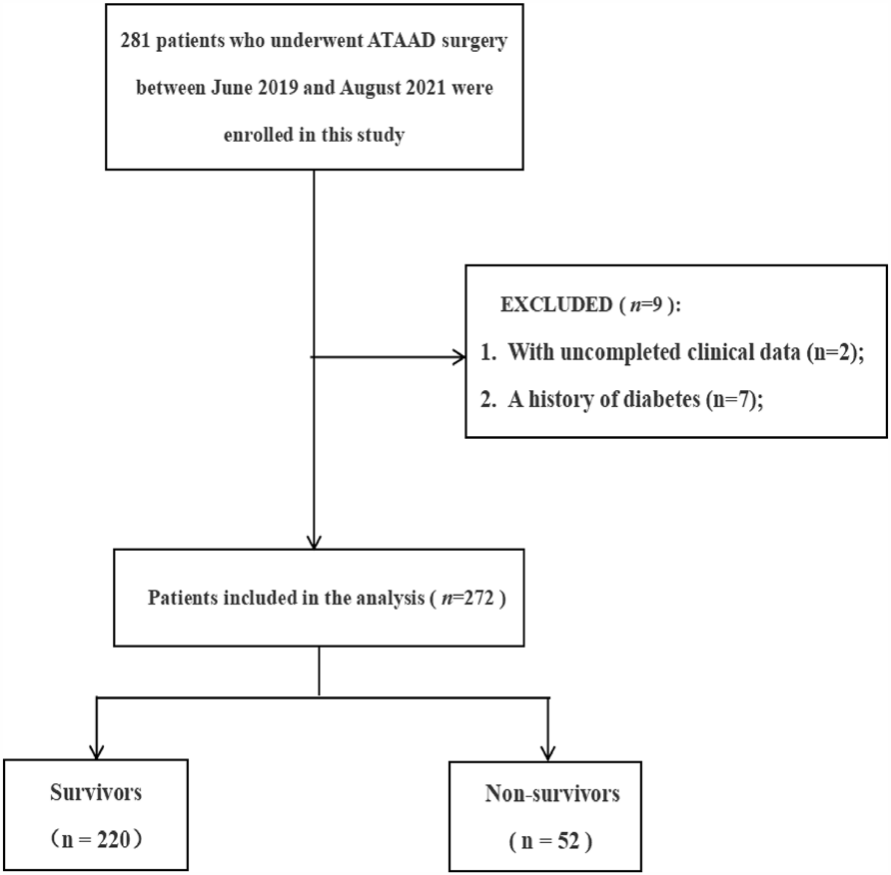
Patient flow chart of the cohort. *ATAAD* Acute type A aortic dissection.

From June 2019 to August 2021, a total of 272 patients diagnosed with ATAAD by CT and magnetic resonance imaging (MRI) were admitted to Union Hospital of Fujian Medical University. The inclusion criteria were adult patients (≥ 18 years old) undergoing ATAAD surgery. Exclusion criteria: (1) a history of diabetes; (2) patients with intravenous and oral potassium; (3) patients with a history of chronic kidney disease; (4) single or multiple organ failure was present on admission; (5) sepsis, or cancer at admission; (6) patients without full medical records. All patients were admitted to the cardiac surgical ICU and given routine ICU care, including sedation, analgesia, and oxygen. The specific screening process is shown in Fig. 1.

For ATAAD patients diagnosed by CTA, in terms of surgical options, in addition to Bentall procedure was performed in 15% of ATAAD patients, and 85% of the patients underwent implantation of modified triple-branched stent graft for descending aorta replacement.

### Data collection

The clinical data of all patients were retrospectively collected by searching clinical electronic database. Socio-demographic characteristics were collected, including age, sex, BMI, past history, and living habits. Medical records and laboratory findings were collected, such as temperature, blood pressure and heart rate at admission, blood tests and biochemical tests.

### Variables definition

GPR was calculated by serum glucose (mmol/L)/potassium (mmol/L) in the first blood test results after admission^13^. In-hospital mortality was the primary end point, and secondary end points were other postoperative complications during hospitalization, including acute renal insufficiency, acute liver insufficiency, gastrointestinal hemorrhage, MODS, cerebral infarction, and neurological complications. The duration of ICU stay was also recorded. Acute renal insufficiency was defined as increasing in SCr by 50% within 7 days or increase in SCr by 0.3 mg/dL (26.5 μmol/L) within 2 days or oliguria for ≥ 6 h^17^. Acute liver insufficiency was defined as postoperative ALT (0–46 IU/L) and/or AST (0–46 IU/L) exceeded the normal value; TBIL (2–22 umol/L) and/or DBIL (0–5.9 umol/L) were elevated^31,32^. Gastrointestinal hemorrhage was defined in this study as the presence of hematemesis and/or blackness, with or without dizziness, palpitations, pallor, rapid heart rate, decreased blood pressure, and other signs of peripheral circulatory failure^33^. Cerebral infarction is defined as neurological dysfunction caused by focal cerebral or retinal ischemia, with clinical symptoms lasting less than 24 h or less^34^. MODS was defined as a sequential organ failure score > 6 at least 48 h (2 consecutive days or more) after admission^18^.

### Data analysis

All data were analyzed using IBM SPSS® 26.0. All patients were divided into high and low groups according to the median GPR, and the characteristics of patients with different GPR levels were analyzed and compared. Measurement data were expressed as mean and standard deviation (SD), and Shapiro–Wilk test was used to test the normality of continuous variables. When normal distribution is satisfied, it is described by mean and standard deviation. When normal distribution is not satisfied, the median and interquartile range are used. For normally distributed data, Student's t test was used to compare two independent samples. For the comparison of non-normally distributed data, the Mann–Whitney U test was used. Enumeration data were expressed as numbers and percentage values (%), and differences of categorical variables were compared by chi-square test. When the chi-square test conditions were not met, Fisher exact test was used, and values of *P* < 0.05 (two-sided) were considered significant. First, univariate logistic regression analysis was used to determine the potential risk factors for in-hospital mortality (*P* < 0.1), and then multivariate logistic regression analysis was used to confirm that the previously significant variables were independent factors (*P* < 0.05).

### Ethical declarations

This study was approved by the Ethics Committee of Union Hospital affiliated to Fujian Medical University (No. 2019KY019). We informed consent was obtained from all subjects and their legal guardian(s).

## Results

From June 2019 to August 2021, a total of 281 patients were included in the study, excluding 2 patients with incomplete medical records and 7 patients with diabetes, and 272 subjects were finally included in the study (Fig. 1). There were 220 survivors and 52 deaths. The causes included MODS (44 cases), dissection ruptured again after operation (5 cases), lung infection lead to respiratory failure (3 cases). The mortality rate of ATAAD patients was 19.1%. As shown in Table 1, there were 201 males (73.9%) and 71 females (26.1%) in this study. The average age of the patients was 53.60 ± 12.57. And 188 patients with hypertension (69.1%).

**Table 1:** Preoperative baseline data of patients grouped by GPR ratio (n = 272).

Variables	Total (n = 272)	GPR		*P*
		Low ≤ 1.74 (n = 137)	High > 1.74 (n = 135)	
Age (years), mean (SD)	53.60 ± 12.57	54.00 ± 12.40	55.68 ± 14.07	0.371
Male, n (%)	201 (73.9)	102 (74.5)	99 (73.3)	0.834
BMI (kg/m^2^), mean (SD)	24.67 ± 2.30	24.54 ± 2.25	24.78 ± 2.35	0.389
Hypertension, n (%)	188 (69.1)	93 (67.9)	95 (70.4)	0.573
Coronary artery disease, n (%)	9 (3.3)	4 (2.9)	5 (3.7)	0.718
Smoker, n (%)	113 (41.5)	63 (46.0)	50 (37.0)	0.134
Drinker, n (%)	63 (23.2)	31 (22.6)	32 (23.7)	0.833
Heart rate, mean (SD)	80.22 ± 15.05	80.92 ± 13.86	79.50 ± 16.19	0.439
Systolic BP (mm/Hg), mean (SD)	141.14 ± 31.96	135.34 ± 29.79	147.04 ± 33.11	0.002*
Diastolic BP (mm/Hg), mean (SD)	76.56 ± 17.03	73.58 ± 16.44	79.58 ± 17.14	0.003*
PP (mm/Hg), mean (SD)	65.02 ± 23.74	61.82 ± 21.85	68.26 ± 25.18	0.025*
Operating time (min), median (IQR)	312.00 (280.00–343.75)	312.84 (282.00–350.50)	312.84 (275.00–339.00)	0.374
Postoperative LVEF (%), median (IQR)	61.83 (60.00–65.60)	61.98 (59.45–65.35)	62.20 (60.20–66.00)	0.200

*BMI* body mass index, *BP* blood pressure, *PP* pulse pressure, *LEVF* left ventricular ejection fraction, *SD* standard deviation, *IQR* interquartile range.

*Significant difference at *P* value < 0.05.

### Preoperative clinical characteristics of different GPR groups

Baseline characteristics of GPR grouped by median are shown in Tables 1 and 2. The median GPR was 1.74 (1.40–2.06). Compared with patients with higher GPR level, the mean values of systolic blood pressure, diastolic blood pressure and pulse pressure difference in the low GPR group were significantly lower than those in the high GPR group (*P* < 0.05). There were significant differences in platelet and GPR between the two groups (*P* < 0.05). There was no significant difference in leukocyte, neutrophil, hemoglobin, albumin, urea and serum creatinine groups (as shown in Table 2). In Table 3, in-hospital mortality was significantly higher in the high GPR group than in the low GPR group (24.4% vs 13.9%: *P* = 0.027). The incidence of renal failure in the low GPR group was significantly higher than that in the high GPR group (26.3% vs 14.8%; *P* = 0.019). There was no significant difference in the duration of ICU stays between low GPR group and high GPR group (*P* > 0.05).

**Table 2:** Preoperative laboratory test data of the patients grouped by GPR (n = 272).

Variables	Total (n = 272)	GPR		*P*
		Low ≤ 1.74 (n = 137)	High > 1.74 (n = 135)	
Neutrophil (× 10^9^/L), mean (SD)	11.61 ± 8.20	12.08 ± 11.02	11.14 ± 3.50	0.340
Hb (g/L), median (IQR)	131.50 (120.00–144.00)	131.00 (118.00–144.00)	132.00 (122.00–144.00)	0.282
PLT (× 10^9^/L), median (IQR)	179.50 (144.25–218.00)	184.00 (154.50–235.00)	178.00 (133.00–206.00)	0.005*
Albumin (g/L),median (IQR)	38.52 (35.43–41.00)	38.00 (34.60–41.00)	38.70 (36.80–41.00)	0.068
WBC (× 10^9^/L), median (IQR)	12.56 (9.76–15.14)	12.57 (9.44–15.39)	12.59 (10.00–14.66)	0.689
Lac (mmol/L), median (IQR)	5.85 (3.23–6.00)	5.91 (2.75–5.95)	5.91 (3.60–6.20)	0.608
Urea (mmol/L), median (IQR)	7.60 (5.30–8.42)	7.40 (5.1–9.45)	8.10 (5.6–8.42)	0.653
SCr (umol/L), median (IQR)	90.00 (68.00–118.00)	93.00 (68.70–129.00)	88.00 (65.00–118.70)	0.058
GPR, median (IQR)	1.74 (1.40–2.06)	1.40 (1.23–1.55)	2.06 (1.86–2.39)	< 0.001*

*Hb*, hemoglobin, *PLT* platelet, *WBC* white blood cell, *SD* standard deviation, *IQR* interquartile range, *GPR* glucose-potassium ratio, *Lac* lactic acid, *SCr* serum creatinine.

*Significant difference at *P* value < 0.05.

**Table 3:** The clinical data of intraoperative patients were grouped by GPR (n = 272).

Variables	Total (n = 272)	GPR		*P*
		Low ≤ 1.74 (n = 137)	High > 1.74 (n = 135)	
Operating time (min), median (IQR)	313.0 (280.0, 344.0)	313.0 (282.0, 350.0)	310.0 (275.0–339.0)	0.340
CPB time (min), median (IQR)	156.0 (129.0, 172.0)	156.0 (135.0, 172.0)	156.0 (125.0, 173.0)	0.408
Crossclamp time (min), median (IQR)	75.0 (51.0, 85.0)	61.5 (45.3, 98.0)	70.0 (48.0, 80.0)	0.081
Duration of MV (h), median (IQR)	41.0 (16.7, 79.5)	41.0 (17.0, 79.5)	41.0 (16.0, 81.0)	0.782

*CPB* cardiopulmonary bypass, *MV* mechanical ventilation

*Significant difference at *P* value < 0.05.

### Intraoperative clinical characteristics of different GPR groups

As shown in Table 3, there was no statistically significant difference in operation duration, CPB duration, and aortic occlusion duration between the euhydration group and the impending and current dehydration group (*P* > 0.05).

### Postoperative clinical outcome between different GPR groups

**Figure 2:**
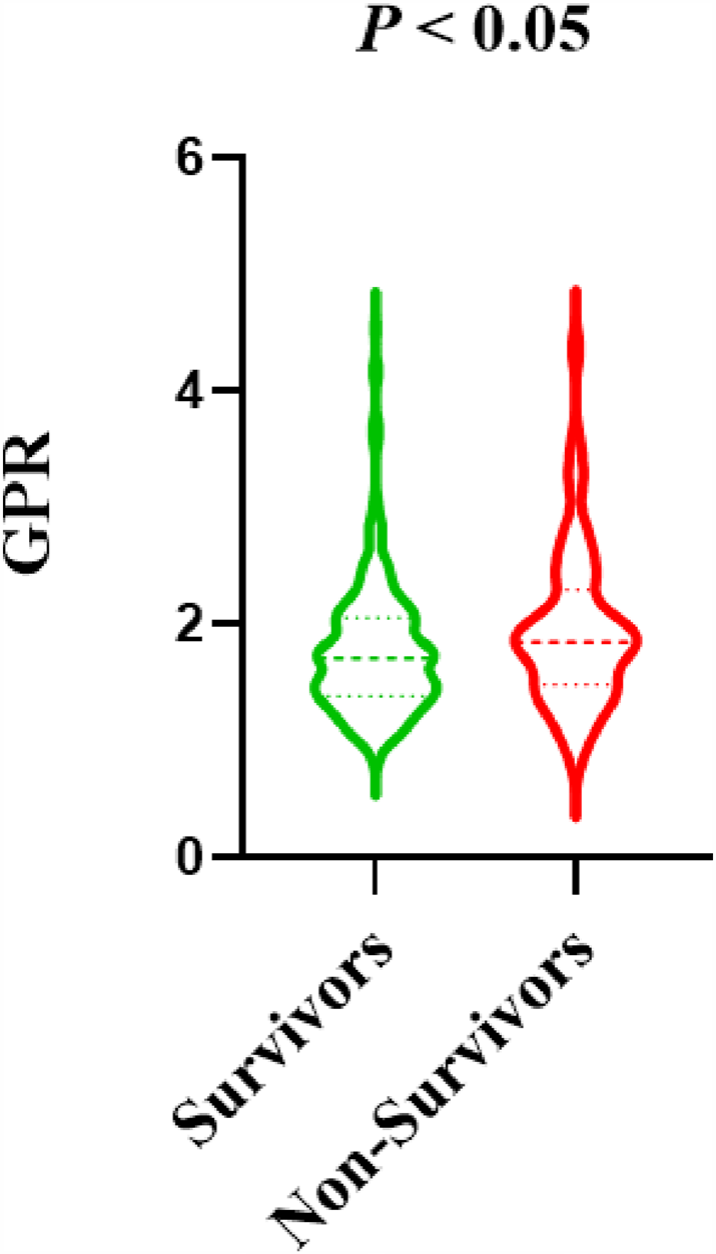
The violin plots for GPR grouping in surviors group and non-survivors group. *GPR* glucose-potassium ratio. Significant difference at *P* value < 0.05.

As shown in Table 4 and Fig. 2, postoperative in-hospital mortality was more common in patients with ATAAD in the high GPR group (> 1.74) (24.4% vs 13.9%; *P* = 0.027). Patients in the low GPR group (≤ 1.74) were more likely to develop acute renal insufficiency than those in the high GPR group (> 1.74) (26.3% vs 14.8%; *P* = 0.019). There was no significant difference in the incidence of acute liver insufficiency, gastrointestinal bleeding, cerebral infarction, MODS and ICU stay between the two groups (*P* > 0.05). There were no significant differences in postoperative blood glucose, potassium, and GPR between the two groups (As shown in Supplementary Tables S1 and S2).

**Table 4:** In-hospital outcomes after ATAAD surgery were grouped by GPR (n = 272).

Variables	Total (n = 272)	GPR		*P*
		Low ≤ 1.74 (n = 137)	High > 1.74 (n = 135)	
Acute renal insufficiency, n (%)	56 (20.6)	36 (26.3)	24 (14.8)	0.019*
Acute liver insufficiency, n (%)	44 (16.2)	26 (20.0)	18 (13.3)	0.206
Gastrointestinal hemorrhage, n (%)	10 (3.7)	5 (3.6)	5 (3.7)	0.981
Cerebral infarction, n (%)	11 (4.0)	5 (3.6)	6 (4.4)	0.383
MODS, n (%)	4 (1.5)	2 (1.5)	2 (1.5)	0.982
In-hospital mortality, n (%)	52 (19.1)	19 (13.9)	33 (24.4)	0.027*
ICU stays (d), median (IQR)	3.75 (1.80–6.78)	3.44 (1.74–6.63)	3.97 (1.92–6.80)	0.493

*GPR* glucose-potassium ratio, *ICU* intensive care unit, *IQR* interquartile range, *MODS* multiple organ dysfunction syndrome.

*Significant difference at *P* value < 0.05.

### Multivariate logistic regression analysis results

Logistic regression was used to analyze the independent risk factors of in-hospital mortality in ATAAD patients. Univariate logistic regression analysis showed that GPR, LVEF, Lac, operating time and serum albumin were associated with in-hospital death (*P* < 0.05), which may be risk factors for in-hospital death in patients with type ATAAD (as shown in Table 5). Age, creatinine and WBC were not correlated with in-hospital mortality (*P* > 0.05). After excluding the influence of other factors, multivariate logistic regression analysis showed high GPR (> 1.74) (AOR 4.70, 95% confidence interval (CI) 2.13–10.40; *P* =  < 0.001), lactic acid (AOR 1.14, 95% CI 1.03–1.26; P = 0.009), smoking (AOR 2.45, 95% CI 1.18–15.07; *P* = 0.039), receiving mechanical ventilation (AOR 9.47, 95% CI 4.00–22.38; *P* =  < 0.001) was independent risk factor for in-hospital death in ATAAD patients, serum albumin (AOR 0.90, 95% CI 0.83–0.98; *P* = 0.014) was a protective factor for in-hospital prognosis.

**Table 5:** Determine the risk factors of in-hospital mortality by logistic regression analysis.

Independent variables	Univariate analysis			Multivariate analysis		
	OR	95% CI	*P*	OR	95% CI	*P*
GPR (> 1.74)	2.009	1.077–3.748	0.028*	4.702	2.125–10.401	< 0.001*
Preoperative LEVF (%)	0.953	0.917–0.991	0.016*	0.970	0.926–1.016	0.157
Lac (mmol/L)	1.116	1.037–1.202	0.004*	1.136	1.028–1.256	0.009*
Operating time (min)	1.006	1.002–1.010	0.005*	1.008	1.002–1.014	0.028*
Serum albumin (g/L)	0.918	0.860–0.980	0.010*	0.904	0.833–0.981	0.014*
Smokers (n)	1.853	1.008–3.407	0.047*	2.450	1.183–5.074	0.039*
Mechanical ventilation (n)	4.457	2.317–8.575	< 0.001*	9.470	4.007–22.382	< 0.001*

*CI* confidence interval, *GPR* glucose-potassium ratio, *LEVF* left ventricular ejection fraction, *Lac* lactic acid, *CPB* cardiopulmonary bypass.

*Significant difference at *P* value < 0.05.

## Discussion

ATAAD is one of the most common diseases in the emergency treatment of cardiovascular surgery. Early detection of risk factors in patients with ATAAD can effectively reduce the risk of death. This study is the first to evaluate the relationship between GPR at admission and in-hospital mortality in patients with ATAAD. The results showed that ATAAD patients with high levels of GPR on admission had an increased risk of postoperative hospitalization death. Higher levels of GPR remained independently associated with in-hospital mortality after adjusting for other factors.

We compared the differences in in-hospital outcomes between the GPR groups and found that in-hospital mortality was significantly higher in ATAAD patients with higher GPR than in patients with lower GPR. In this study, the in-hospital mortality rate for ATAAD patients was 19.1%, which is consistent with other findings^5,19^. Evangelista et al. systematically reviewed the effects of advanced age, cardiac tamponade, hypotension, myocardial ischemia, neurological impairment and other factors on ATAAD^6^, but these factors still cannot meet the requirements of modern clinical practice development.

In recent years, the research on blood sugar and potassium has attracted wide attention from scholars. Several studies have shown that dysglycemia and potassium metabolism are independently associated with major cardiac adverse events (MACE)^7,11,20^. However, it is important to note that the problem of glycaemia does not only refer to persistent high blood glucose, but also to fluctuations in blood glucose, including changes in rise and fall^7,8^. This indicates that the blood glucose index at a single time point has limitations, and it is necessary to combine with other indexes or conduct dynamic blood glucose monitoring. Current studies have proved that GPR is an influential factor for the early prognosis of patients with acute cerebral hemorrhage^21^. It is also an early prognostic factor in death from a number of diseases, including ischemic stroke (IS), traumatic brain injury, and blunt abdominal trauma^13–15^. In these studies, baseline GPR was significantly higher in the group with poor clinical outcomes than in the group with normal GPR, suggesting that patients with high GPR may be highly associated with poor disease outcomes. This also provides a strong theoretical basis for us to study the correlation between GPR and in-hospital mortality in ATAAD patients.

This study also found that high GPR (> 1.74) was an independent predictor of in-hospital death in patients with ATAAD, and remained significant after adjusting for factors such as LEVF, operation time, and mechanical ventilation (*P* < 0.05). The results were consistent with those of Zhou and katipoyplu in patients with severe craniocerebral injury and blunt abdominal trauma. They also found that patients with high GPR had significantly shorter survival times and higher mortality^13,14^. The mechanism for this effect may be that the sympathetic nervous system of patients is stressed after trauma, leading to the production of excessive catecholamine. Glucagon and catecholamine are the main glucagon-regulating hormones involved in hyperglycemia response. After stress, the secretion of glucagon in the body increases, and thus the blood glucose concentration increases^15,22,23^. Xie et al. found that cells in the body are prone to dysfunction and apoptosis due to hyperglycemia^25^. In addition, potassium plays an important role in the cellular activities of the human body, which ensures the continuity of many cell functions. Potassium is primarily stored in cells, and its transport is affected by active uptake of potassium by cell membranes and by the adenosine triphosphatase sodium/potassium pump (Na^+^-K^+^-ATPase). When catecholamine is secreted under stress, it regulates Na^+^-K^+^-ATPase, resulting in a decrease in serum potassium levels^24,25^. Taken together, these findings suggest that GPR has clinical significance in the development of ATAAD and is an early prognostic factor for ATAAD. Active control of hyperglycemia and potassium disturbance on admission may be a new strategy to improve hospitalization survival of ATAAD patients in the future.

Picard et al. showed that urinary sodium/potassium was associated with a faster decline in renal function^26^. At the same time, renal dysfunction was more common in patients with lower GPR (≤ 1.74), and creatinine was significantly higher in patients with higher GPR (*P* < 0.05). The results of this study are consistent with those of the above studies. A large clinical trial has confirmed that hyperkalemia significantly increases the risk of renal dysfunction^27^. These also strongly support the conclusion of this study. Therefore, there may be a reciprocal relationship between potassium level and kidney function. However, there are different voices regarding the relationship between potassium and kidney function in healthy people. It has been suggested that higher dietary potassium intake may protect renal and cardiovascular function in the general population because of the complex regulatory mechanisms of intrinsic plasma potassium^28,29,30^. Of course, the relationship between serum potassium and kidney function needs to be demonstrated and summarized by more prospective studies and randomized clinical trials.

Blood glucose and potassium were derived from serum samples that were routinely tested clinically and easily obtained. As a new biomarker, GPR may help to predict the prognosis of ATAAD patients in the early stage. In clinical practice, the treatment of patients with such disorders is optimized by identifying biochemical variables that reflect subtle changes in the patient's neurological and physical status^14^. At the same time, it may play a certain role in predicting the postoperative decision-making of doctors for patients. As an important supplement of prognostic indicators, it has important clinical significance. In addition, the measure does not cost individuals or health systems more money, adding potential value that could make economic sense if applied in deprived areas in the future.

## Limitations

The study has several limitations. First, our analysis was conducted in a center with limited external validity. A prospective multicenter study is needed in the future to support our conclusions. Second, long-term studies of the effect of GPR on in-hospital mortality in patients with ATAAD are needed to better assess and prevent the formation and development of ATAAD. In addition, due to the retrospective nature of the study, some intraoperative clinical data could not be effectively obtained. Finally, although pre-admission laboratory results were used, which are more likely to reflect the patient's initial state at the time of onset, it is desirable to monitor GPR dynamics in future studies to better understand the underlying mechanisms.

## Conclusions

This study showed that higher GPR was an independent risk factor for in-hospital mortality in patients undergoing ATAAD surgery. Simple and feasible GPR may be an effective preoperative evaluation and screening tool. We suggest that GPR has great potential as a simple and rapid predictor of in-hospital mortality in ATAAD patients.

### Supplementary Information

Supplementary Table S1.

Supplementary Table S2.

## Data Availability

The data used to support the findings of this study are available from the corresponding author upon reasonable request.
