# Supplementary material for: The blood glucose-potassium ratio at admission predicts in-hospital mortality in patients with acute type A aortic dissection
Source: Sci Rep. 2023 Sep 21;13:15707. doi: 10.1038/s41598-023-42827-2 (PMC10514330; doi:10.1038/s41598-023-42827-2)
Supplement: Supplementary file 1 — Supplementary Table S1. [file 41598_2023_42827_MOESM1_ESM.docx]

**Table S1.** Association of DHCA time and CPB postoperative blood glucose and potassium with patients mortality

| **Variables** | **Total (n=272)** | **Survivors (n=220)** | **Non-Survivors (n=52)** | ***P*** |
| --- | --- | --- | --- | --- |
| Glucose (mmol/L), median (IQR) | 7.00 (5.90-8.42) | 3.60 (3.30-4.30) | 3.77 (3.60-4.25) | .390 |
| [Potassium](javascript:;)(mmol/L), median (IQR) | 10.36 (8.73-11.48) | 10.15 (8.50-11.70) | 10.36 (9.80-11.06) | .094 |
| GPR, post-CPB, median (IQR) | 2.54 (2.05-2.98) | 2.54 (2.03-3.00) | 2.54 (2.47-2.73) | .372 |
| DHCA (min), mean (SD) | 7.56±3.33 | 7.56±3.67 | 7.59±3.71 | .489 |

**Note:** *CPB*, cardiopulmonary bypass; *IQR*, interquartile range; *GPR*, glucose-potassium ratio;

*DHCA*, [deep hypothermia circulatory arrest;](javascript:;)^*^Significant difference at *P* value < 0.05.
