# Supplementary material for: The blood glucose-potassium ratio at admission predicts in-hospital mortality in patients with acute type A aortic dissection
Source: Sci Rep. 2023 Sep 21;13:15707. doi: 10.1038/s41598-023-42827-2 (PMC10514330; doi:10.1038/s41598-023-42827-2)
Supplement: Supplementary file 2 — Supplementary Table S2. [file 41598_2023_42827_MOESM2_ESM.docx]

**Table S2.** Association of DHCA time and CPB postoperative blood glucose and serum potassium and renal insufficiency in patients

| **Variables** | **Total (n=272)** | **No [insufficiency](javascript:;) (n=216)** | **[insufficiency](javascript:;) (n=56)** | ***P*** |
| --- | --- | --- | --- | --- |
| Glucose (mmol/L), median (IQR) | 7.00 (5.90-8.42) | 6.80 (5.87-8.63) | 7.00 (5.62-8.40) | .866 |
| [Potassium](javascript:;)(mmol/L), median (IQR) | 10.36 (8.73-11.48) | 10.00 (8.43-11.70) | 10.40 (8.34-12.80) | .537 |
| GPR, post-CPB, median (IQR) | 2.54 (2.05-2.98) | 2.54 (2.06-2.93) | 2.54 (2.01-3.26) | .883 |
| DHCA (min), mean (SD) | 7.56±3.33 | 7.55±3.40 | 7.61±3.37 | .223 |

**Note:** *CPB*, cardiopulmonary bypass; *IQR*, interquartile range;*GPR*, glucose-potassium ratio;

*DHCA*, [deep hypothermia circulatory arrest; SD, standard deviation;](javascript:;)^*^Significant difference at *P* value < 0.05.
